# Whole-Genome Sequencing Reveals Population Structure, Genetic Diversity, and Selection Signatures in Kazakh Dromedary and Bactrian Camels

**DOI:** 10.3390/ani16172793

**Published:** 2026-09-05

**Authors:** Zhannur Niyazbekova, Cai-Yue Gao, Nursultan Makhanbetuly, Kuanysh Kassen, Yuan Xu, Bekzat Baimirzayev, Huanhuan Zhang, Zhadyra Muslimova, Kanat Orynkhanov, Yessengali Ussenbekov, Fengting Bai, Junyan Wang, Hasan Baneh, Yelaman Serikov, Pilong Liu, Yu Jiang

**Affiliations:** 1 Faculty of Veterinary Medicine and Zooengineering, Kazakh National Agrarian Research University, Almaty 050000, Kazakhstan; nursultan.makhanbetuly@kaznaru.edu.kz (N.M.); baimyrzaev.bekzat@kaznaru.edu.kz (B.B.); muslimova.zhadyra@kaznaru.edu.kz (Z.M.); kanat.orynkhanov@kaznaru.edu.kz (K.O.); yessengali.ussenbekov@kaznaru.edu.kz (Y.U.); 2 College of Animal Science and Technology, Northwest A&F University, Yangling 712100, China; gaocaiyue@nwafu.edu.cn (C.-Y.G.); xuyuan@nwafu.edu.cn (Y.X.); zhanghuanhuan0914@126.com (H.Z.); bft990914@163.com (F.B.); wangjunyan1996@foxmail.com (J.W.); liu.pl@nwafu.edu.cn (P.L.); 3 Plant Biotechnology Center, Kazakh National Agrarian Research University, Almaty 050000, Kazakhstan; kassenkuanysh12@gmail.com; 4 Center for Bio- and Medical Technologies, Skolkovo Institute of Science and Technology (Skoltech), Moscow 121205, Russia; hasanbaneh@gmail.com; 5 Faculty of Information Technology and Artificial Intelligence, Al-Farabi Kazakh National University, Almaty 050000, Kazakhstan; yelaman1x9@gmail.com

**Keywords:** whole-genome sequencing, camel, population genomics, genetic diversity, *MC4R*, *RYR1*, environmental adaptation

## Abstract

Understanding the genomic basis of environmental adaptation is essential for the conservation and genetic improvement of domestic camels. In this study, we investigated the population structure, genetic diversity, and genomic variation potentially associated with environmental adaptation of Kazakh dromedary and Bactrian camels using whole-genome sequencing. Whole-genome sequencing data were generated for Kazakh camels (15 dromedaries and 16 Bactrian camels) and integrated with 131 publicly available genomes representing camel populations from the Arabian Peninsula, Iran, Xinjiang, Inner Mongolia, and Mongolian wild camels. Population structure, genetic diversity, and genome-wide selection were evaluated using principal component analysis, ADMIXTURE, nucleotide diversity, linkage disequilibrium, runs of homozygosity, genomic inbreeding (FROH), and selection scans based on *F*_ST_, θπ ratio, and XP-EHH. Population genomic analyses revealed clear differentiation between dromedary and Bactrian camels, whereas Kazakh camel populations exhibited higher nucleotide diversity (θπ = 1.307–1.551 × 10^−3^), and lower genomic inbreeding (median FROH: 0.037–0.056) than Arabian populations. Genome-wide selection analyses identified *MC4R* as the prominent candidate gene in Kazakh dromedaries and *RYR1* as a prominent candidate gene in Kazakh Bactrian camels. Functional enrichment analyses highlighted pathways related to energy metabolism, thermogenesis, calcium signaling, skeletal muscle function, mitochondrial activity, and oxidative stress response. These findings provide new insights into genomic variation potentially associated with environmental adaptation in Kazakh camels and offer valuable genomic resources for future conservation, breeding, and evolutionary studies.

## Simple Summary

Camels are well adapted to harsh environments and are important for livestock production in arid and semi-arid regions. However, the genetic characteristics of Kazakh camel populations remain poorly understood. In this study, we analyzed whole-genome sequences of Kazakh dromedary and Bactrian camels and compared them with camel populations from other regions. We found that Kazakh camels exhibited relatively high genetic diversity and lower levels of inbreeding than several comparison populations. We also identified candidate genes potentially associated with environmental adaptation, including genes related to energy metabolism, thermoregulation, and muscle function. The findings improve our understanding of the origin, breeding history, and environmental adaptation of Kazakh camels. The results may support the conservation of local camel genetic resources and help develop more effective breeding programs for productivity, resilience, and sustainable camel farming.

## 1. Introduction

Camels (*Camelus* spp.) are among the most important livestock species inhabiting arid and semi-arid regions, providing milk, meat, fiber, transportation, and draft power while supporting the livelihoods of pastoral communities [1,2]. Their exceptional tolerance to heat, dehydration, nutritional stress, and extreme environmental conditions has enabled them to thrive in ecosystems that are unsuitable for most domestic livestock [3,4,5]. These unique physiological and metabolic adaptations have attracted increasing scientific interest in the genetic mechanisms underlying environmental adaptation and resilience.

Recent advances in whole-genome sequencing (WGS) have substantially improved our understanding of camel evolution, domestication, migration, and environmental adaptation [6]. Genome-wide analyses have reconstructed the demographic history of domestic camels, clarified the evolutionary relationships among dromedaries, domestic Bactrian camels, and wild camels, and revealed historical admixture and introgression among geographically distinct populations [7,8].

Previous genomic studies have identified candidate genes associated with important biological traits in different camel populations. These include *ESR1* associated with reproductive traits in Saudi Arabian dromedaries [9], *MYO3A*, and *TMOD3* associated with growth in Iranian dromedaries [10], and heat shock proteins (HSPs) involved in heat-stress responses in Arabian dromedaries [11]. In addition, selection signature analysis of Iranian dromedary and Bactrian camels identified candidate genes potentially associated with environmental adaptation, including *ZNF516* and *SLC12A1* [12]. Furthermore, comparative genomic analyses of camel populations from the Arabian Peninsula, Iran, Mongolia, China and Central Asia have revealed substantial variation in nucleotide diversity, linkage disequilibrium, genomic inbreeding, and selective sweep patterns reflecting differences in domestication history, breeding practices, natural selection, and local environmental adaptation [8,13].

Kazakhstan occupies a unique geographical position within the historical distribution of domestic camels and is one of the few countries where indigenous dromedary and Bactrian camels are traditionally raised together [14]. The country’s sharply continental climate, characterized by extremely hot summers, long cold winters, low precipitation, and highly seasonal pasture availability, has imposed strong environmental pressures that have likely shaped the genomic architecture of local camel populations [15]. Kazakhstan has a long history of camel breeding and hybridization, with crosses between dromedaries and Bactrian camels developed to improve milk production, body size, and adaptation to harsh climatic conditions [16,17]. These hybrids represent an important component of the national camel industry and have been extensively utilized in breeding programs throughout Central Asia.

Despite the economic and cultural importance of camels in Kazakhstan, genomic studies remain limited. Earlier investigations mainly relied on microsatellite markers, mitochondrial DNA, and candidate-gene analyses to evaluate genetic diversity and breed relationships [18,19,20,21]. More recently, Amandykova et al. [22] reported the first whole-genome sequencing analysis of hybrid camels bred in Kazakhstan by comparing five hybrid genomes with publicly available dromedary, Bactrian, and wild camel genomes. Although this study demonstrated the utility of WGS for characterizing the genomic composition of hybrid camels, it focused exclusively on hybrid animals and did not comprehensively investigate the genomic diversity, population structure, or adaptive evolution of indigenous Kazakh dromedary and Bactrian camels. Moreover, no genome-wide comparative study has systematically evaluated the genetic relationships between indigenous Kazakh camel populations and geographically diverse camel populations to identify genomic regions associated with local environmental adaptation. Consequently, the genomic basis of adaptation in indigenous Kazakh camels remains largely unknown.

In the present study, we performed the first comprehensive population genomic analysis of indigenous Kazakh dromedary and Bactrian camels using whole-genome sequencing. We generated WGS data for 31 indigenous Kazakh camels and integrated these data with 131 publicly available genomes representing camel populations from the Arabian Peninsula, Iran, Xinjiang, Inner Mongolia, and Mongolian wild camels. The objective of this study was to characterize the population structure and genetic diversity of indigenous Kazakh camels and to identify genomic regions and candidate genes potentially associated with environmental adaptation. We hypothesized that Kazakh camel populations would exhibit distinct patterns of genetic diversity and genomic selection signatures compared with geographically distinct camel populations. Using complementary population genomic approaches, including analyses of population genetic structure, genetic diversity, genomic inbreeding, and selection signals, we investigated the genetic relationships and adaptive genomic architecture of Kazakh camel populations. Functional enrichment analyses were performed to identify biological pathways associated with environmental adaptation. Our findings provide new insights into the genetic diversity and adaptive mechanisms of indigenous Kazakh camels and establish valuable genomic resources for future conservation, molecular breeding, and sustainable management of camel genetic resources.

## 2. Materials and Methods

### 2.1. Sample Collection

Blood samples were collected from 16 Bactrian camels (*Camelus bactrianus*; KAZ_B) and 15 dromedary camels (*Camelus dromedarius*; KAZ_D) from private farms in the Kyzylorda, Turkestan, and Almaty regions of southern Kazakhstan (Table S1 and Figure S1). All animal handling and sample collection procedures were conducted in accordance with the protocol approved by the Ethics Committee of Kazakh National Agrarian Research University (Protocol No. 02, approved on 7 October 2025). All procedures complied with institutional guidelines for the care and use of animals. No adverse health effects were observed during or after blood collection, and all animals remained healthy throughout the study. Prior to blood sample collection, verbal permission for sampling was obtained from the farm owners.

Genomic DNA was extracted from whole-blood samples using the standard phenol-chloroform method [23]. Paired-end sequencing libraries with an average insert size of 318 bp were constructed for each individual and sequenced with a 150 bp paired-end configuration on the Illumina NovaSeq X Plus platform (Illumina, San Diego, CA, USA).

To increase the geographic representation of camel populations, publicly available whole-genome sequencing datasets were incorporated into the analyses. These datasets comprised 131 individuals, including Mongolian wild Bactrian camels (Wild, *n* = 11), Xinjiang Junggar Bactrian camels (XJ, *n* = 17), Inner Mongolian Bactrian camels (NMG, *n* = 17), previously reported Kazakh Bactrian camels (KAZ_B, *n* = 6), Iranian dromedaries (Iran, *n* = 6), Saudi Arabian dromedaries (Saudi, *n* = 19), Kuwaiti dromedaries (Kuwait, *n* = 13), Omani dromedaries (Oman, *n* = 37), and Qatari dromedaries (Qatar, *n* = 5). In total, whole-genome sequencing data from 162 camels were included in the subsequent population genomic analyses.

### 2.2. Genome Data Processing

Raw sequencing reads were subjected to quality control using fastp v0.23.4 [24]. Reads were removed if (1) 10% or more of the bases were uncertain (N); (2) 40% or more of the bases were of low quality (Qphred ≤ 20); (3) the read length was shorter than 15 bases; or (4) sequence complexity was less than 30%. Clean reads were aligned to the CamDro3 reference genome (https://www.ncbi.nlm.nih.gov/datasets/genome/GCF_000803125.2/ (accessed on 2 March 2026)) using BWA-MEM v0.7.5a-r405 [25]. The same reference genome and alignment pipeline were applied to all populations, including dromedary, domestic Bactrian, and wild Bactrian camels, to maintain a consistent genomic coordinate system and minimize technical heterogeneity among datasets. As this study primarily focused on autosomal population genetic variation, only the 36 autosomes were retained for subsequent analyses, while sex chromosomes and unplaced scaffolds were excluded. Sequence Alignment/Map (SAM) files were converted to Binary Alignment/Map (BAM) format, sorted, and indexed using SAMtools v1.18 [26]. PCR duplicates were identified and removed using Picard v2.21.2 (https://broadinstitute.github.io/picard/ (accessed on 9 March 2026)).

Single-nucleotide polymorphisms (SNPs) were identified using the HaplotypeCaller module of implemented GATK v4.3.0.0 [27]. Individual gVCF files were merged using CombineGVCFs, followed by joint genotyping with GenotypeGVCFs. SNPs were filtered using the following hard-filtering criteria: QD < 2.0, MQ < 40.0, QUAL < 40.0, FS > 60.0, MQRankSum < −12.5, and ReadPosRankSum < −8.0. Additional filtering was performed using BCFtools v1.17 [26] to retain biallelic SNPs with a missing genotype rate ≤ 10%. Mapping rate, effective sequencing depth, genotype missingness, and reference/alternative allele balance were assessed separately for each population and are reported in Table S3. Separate SNP datasets were then generated according to the requirements of downstream analyses. For population structure analyses, including principal component analysis (PCA), neighbor-joining (NJ) tree construction, and ADMIXTURE, SNPs with a minor allele frequency (MAF ≥ 0.01) were retained. For nucleotide diversity (θπ), ROH, FROH, and selection analyses (*F*_ST_, XP-EHH, and θπ ratio), no additional MAF filtering was applied to retain rare variants.

### 2.3. Population Genetic Structure Analysis

Pairwise genetic relatedness among all individuals was estimated using KING v2.3.1 [28], with a kinship coefficient > 0.177 used to identify first-degree relatives. For each detected first-degree relative pair, one individual was retained and the other was excluded. All 162 camel samples included in this study were retained after relatedness filtering, and pairwise kinship estimates are provided in Table S4. Population genetic structure was investigated using WGS data from 162 camels. Following variant filtering, 7,757,093 high-quality SNPs with a MAF ≥ 0.01 and a Hardy–Weinberg equilibrium (HWE) *p*-value ≥ 1 × 10^−6^ were retained for subsequent population genetic structure analyses. To minimize the effects of linkage disequilibrium (LD), SNP pruning was performed using PLINK v1.90 [29] with the parameter --indep-pairwise 50 10 0.4, resulting in 1,534,519 LD-pruned SNPs for subsequent analyses. Population genetic structure was assessed using PCA, NJ phylogenetic analysis, and ADMIXTURE.

Principal component analysis (PCA) was conducted using the smartPCA program implemented in EIGENSOFT v5.0 [30]. Pairwise genetic distances were calculated using PLINK v1.90 [29], and a NJ phylogenetic tree was constructed using MEGA v6.0 [31]. The resulting phylogenetic tree was visualized using the Interactive Tree of Life (iTOL) web server [32].

Population genetic structure was further inferred using ADMIXTURE v1.3.0 [33]. To reduce potential bias arising from unequal population sample sizes, the largest population, Oman (*n* = 37), was randomly subsampled to 20 individuals, while all individuals from the remaining populations were retained, resulting in a sample-size-adjusted dataset of 145 individuals: Iran (*n* = 6), KAZ_B (*n* = 22; 16 newly sequenced and 6 publicly available individuals), KAZ_D (*n* = 15), Kuwait (*n* = 13), NMG (*n* = 17), Oman (*n* = 20), Qatar (*n* = 5), Saudi (*n* = 19), Wild (*n* = 11), and XJ (*n* = 17). Subsampling was independently repeated 20 times using different random seeds. For each replicate the number of ancestral populations (*K*) was evaluated from *K* = 2 to *K* = 9, and the optimal value of *K* was determined based on the lowest mean cross-validation (CV) error across the 20 replicates. Individual ancestry proportions are provided in Table S5.

### 2.4. Population Genetic Diversity Analysis

Linkage disequilibrium (LD) decay was evaluated using PopLDdecay v3.42 [34] with default parameters, and LD decay curves were generated across increasing physical distances. Nucleotide diversity (θπ) was calculated for each population using VCFtools v0.1.13 [35] with a sliding window size of 10 kb and a step size of 2 kb (--window-pi 10,000--window-pi-step 2000). The analysis was based on the SNP-only VCF dataset filtered for a missing genotype rate ≤ 10%; therefore, invariant sites were not explicitly included. Missing genotypes were excluded from pairwise calculations, and θπ was estimated using available genotype information within each window.

Runs of homozygosity (ROH) were identified using the --homozyg module implemented in PLINK v1.90 [29] with parameters --homozyg-snp 50, --homozyg-kb 500, and --homozyg-gap 1000. The number and total length of ROHs were calculated for each individual and classified into four length categories (0.5–1 Mb, 1–2 Mb, 2–4 Mb, and >4 Mb). The distribution of ROHs among populations was visualized using the ggplot2 package in R v4.3.2.

Individual genomic inbreeding coefficients (FROHs) were estimated by dividing the total length of ROHs for each individual by the total physical length of the 36 autosomes of the CamDro3 reference genome (1,933,916,958 bp), using the same denominator for all individuals. The distribution of FROH values across camel populations was visualized using custom Python v2016Feb01 (Python v3.10.12) scripts. All available individuals within each population were retained for the nucleotide diversity and FROH analyses despite differences in population sample size.

Population-level θπ and FROH values were summarized using medians, with 95% confidence intervals (CIs) estimated using 10,000 bootstrap replicates. Statistical differences between KAZ_D or KAZ_B and other populations were assessed using two-sided Mann–Whitney U tests. *p* values were adjusted for multiple test comparisons using the Benjamini–Hochberg false discovery rate (FDR) correction method, and an FDR-adjusted *p* < 0.05 was considered statistically significant.

### 2.5. Detection of Selection Signals

To identify genomic regions under positive selection in Kazakh camel populations, two population contrasts were analyzed: Kazakh dromedary (KAZ_D) versus Saudi Arabian dromedary (Saudi), and Kazakh Bactrian (KAZ_B) versus Inner Mongolian Bactrian (NMG). Three complementary approaches were employed: the fixation index (*F*_ST_), the nucleotide diversity ratio (θπ ratio), and the cross-population extended haplotype homozygosity (XP-EHH) [36,37].

Genome-wide *F*_ST_ values between populations were calculated using VCFtools v0.1.13 with a 10 kb sliding window and a 2 kb step (--fst-window-size 10000 --fst-window-step 2000). Because selective sweeps are often accompanied by reduced nucleotide diversity, differences in nucleotide diversity between the two populations were compared to infer the direction of selection. Nucleotide diversity for each population was calculated using VCFtools v0.1.13 with a 10 kb sliding window and a 2 kb step size (--window-pi 10000 --window-pi-step 2000). The nucleotide diversity ratio was then calculated as ln[θπ(other population)/θπ(Kazakh population)]. Positive values indicate genomic regions potentially subjected to selective sweeps in the Kazakh populations.

For XP-EHH analysis, haplotypes were first phased using Beagle v5.4 [38], followed by XP-EHH calculation using selscan v1.3.0 with the --xpehh option and default parameters [39]. The mean XP-EHH value of all sites within each 10 kb genomic window was then calculated using a 2 kb step size.

For each of the three statistics, genomic windows were independently ranked according to their respective scores, and the 99th percentile (corresponding to the top 1%) was used as the cutoff threshold for each method. Windows with scores exceeding this threshold were defined as candidate selection windows. Because the three statistics differ in scale and distribution, the cutoff threshold was determined independently for each statistic. The top 1% candidate windows identified by each method were independently annotated using ANNOVAR v2016Feb01 [40], generating three separate candidate-gene lists. Genes supported by at least two of the three methods were considered high-confidence candidate genes and were selected for subsequent analyses. Finally, haplotype structures of representative candidate regions across camel populations were visualized using custom Python scripts.

### 2.6. Allele Frequency Analysis of Missense Variants

Allele frequencies of candidate variants were estimated using VCFtools v0.1.13 [35] based on whole-genome sequencing data from all camel populations. The frequencies of reference and alternative alleles were calculated separately for each population to evaluate the distribution of missense mutations among different camel breeds. The resulting allele frequency data were subsequently used to compare the population-specific distribution of candidate adaptive variants.

### 2.7. GO and KEGG Pathway Enrichment Analysis

Gene Ontology (GO) and Kyoto Encyclopedia of Genes and Genomes (KEGG) pathway enrichment analyses were performed using KOBAS v3.0 [41] for candidate genes jointly identified by at least two selection-signal detection methods. Camel candidate genes were mapped to their human (*Homo sapiens*) orthologues, and the human background gene set implemented in KOBAS was used as the reference. Statistical significance was assessed using the Benjamini–Hochberg correction for multiple testing, and pathways or GO terms with an adjusted *p*-value < 0.05 were considered significantly enriched.

## 3. Results

### 3.1. Genome Resequencing

Whole-genome sequencing was performed for 31 indigenous Kazakh camels, including 15 dromedaries and 16 Bactrian camels. These newly generated data were combined with publicly available whole-genome resequencing datasets retrieved from the NCBI Sequence Read Archive (SRA), representing camel populations from the Arabian Peninsula (Saudi Arabia, Oman, Kuwait, and Qatar), Iran, Xinjiang (XJ), and Inner Mongolia (NMG). Mongolian wild camel individuals were included as an outgroup (Tables S1 and S2). In total, the final dataset comprised 162 camel genomes representing the major domestic camel populations and wild camels.

### 3.2. Genetic Structure and Interactions Within Populations

**Figure 1:**
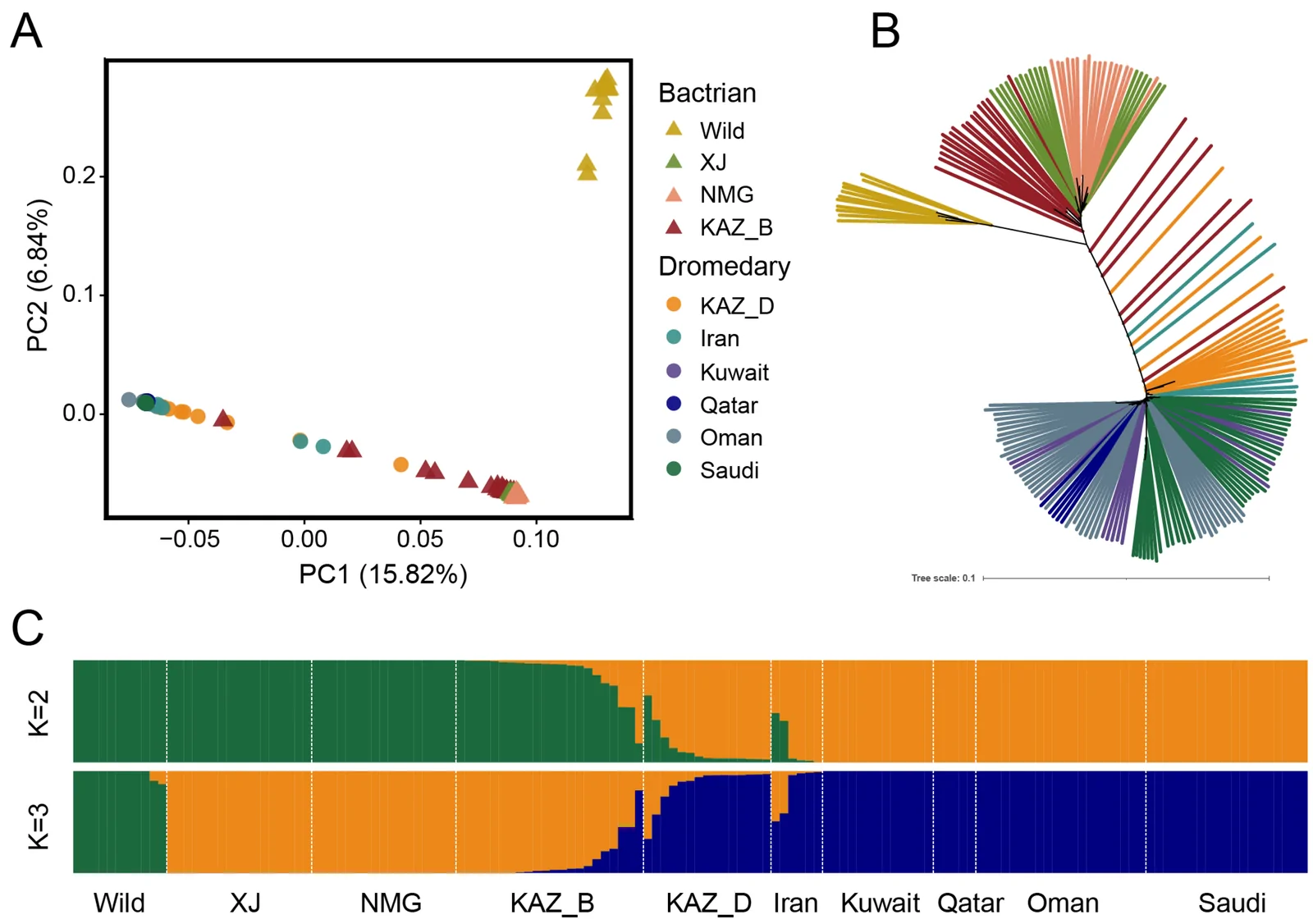
Population genetic structure and phylogenetic relationships among camel populations. (**A**) Principal component analysis (PCA) of camel populations from different geographical regions. (**B**) Neighbor-joining (NJ) phylogenetic tree constructed based on whole-genome SNP data. The scale bar represents the genetic distance between individuals. Colors indicate different geographic populations. (**C**) Population structure analysis of camel populations inferred using ADMIXTURE at *K* = 2 and *K* = 3. Different colors represent distinct ancestral genetic components.

Population structure analysis revealed clear genetic differentiation among the analyzed camel populations. PCA showed that PC1, explaining 15.82% of the total genetic variation, clearly separated dromedaries from Bactrian camels, whereas PC2 (6.84%) further differentiated populations within the dromedaries. Arabian dromedary populations from Saudi Arabia, Oman, Kuwait, and Qatar clustered relatively closely while Kazakh and Iranian dromedaries showed more variable positions. In contrast, domestic Bactrian camels from Xinjiang (XJ), Inner Mongolia (NMG), and Kazakhstan (KAZ_B) clustered closely together. Several individuals from Kazakh dromedaries, Kazakh Bactrian camels, and Iranian dromedaries were positioned between the major clusters along PC1 and PC2, indicating intermediate genetic positions among the analyzed populations (Figure 1A and Figure S2). In order to further examine population differentiation within each species, separate PCAs were performed for dromedary and Bactrian camels. Within dromedaries, the Arabian populations largely overlapped, whereas several KAZ_D and Iranian individuals were differentiated along PC1 and/or PC2 (Figure S3A). Within Bactrian camels, wild camels remained clearly separated from the domestic populations, while XJ and NMG showed substantial overlap. KAZ_B individuals exhibited greater dispersion, with some clustering near XJ and NMG and others differentiated primarily along PC2 (Figure S3B).

The NJ phylogenetic tree showed clustering patterns consistent with the PCA results, with a clear separation between dromedary and Bactrian camel lineages and close genetic relationships among domestic Bactrian populations (Figure 1B). ADMIXTURE analysis further supported these findings (Figure S4). At *K* = 2, dromedary and Bactrian camels formed two distinct genetic clusters, whereas the lowest cross-validation error was observed at *K* = 3 (CV error = 0.266; Figure S5), suggesting that *K* = 3 provided the best-supported clustering solution among the tested *K* values. At *K* = 3, Kazakh dromedary and Kazakh Bactrian camel populations showed more complex patterns of shared genetic ancestry, whereas Arabian dromedary populations exhibited relatively homogeneous genetic structure (Figure 1C). Overall, these results indicate that camel populations in Kazakhstan possess a more complex genetic structure than populations from the Arabian Peninsula, potentially reflecting differences in demographic history, breeding practices, and shared genetic ancestry among geographically neighboring populations.

### 3.3. Genome Variation Patterns

**Figure 2:**
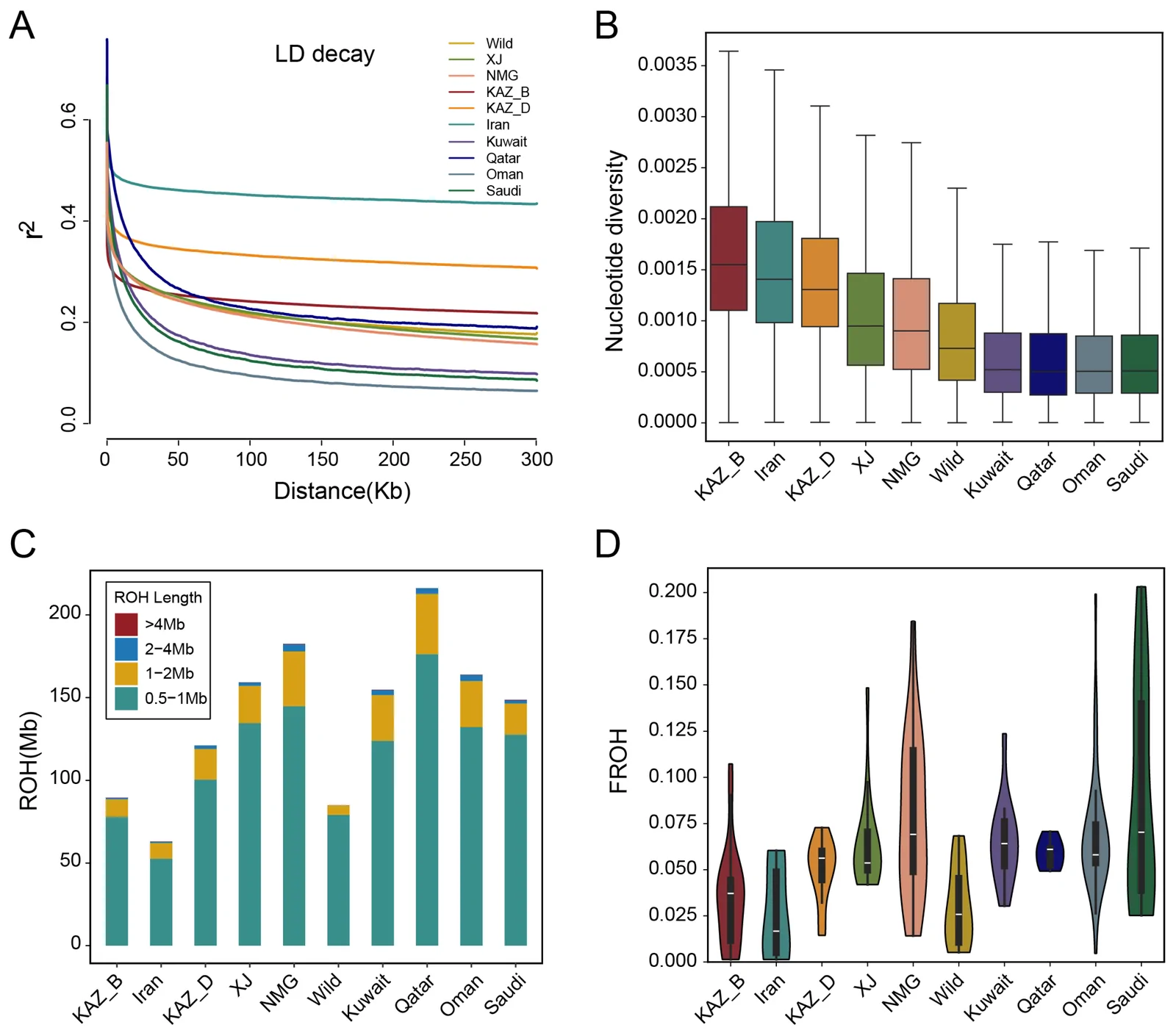
Genetic diversity and inbreeding characteristics of camel populations. (**A**) Linkage disequilibrium (LD) decay across genomic distance, measured by the correlation coefficient (r^2^), in camel populations. (**B**) Nucleotide diversity (θπ) across different camel populations. Boxplots show the median and interquartile range (IQR). (**C**) Number of runs of homozygosity (ROH) detected in the 10 camel populations. (**D**) Distribution of genomic inbreeding coefficient (FROH) estimated from runs of homozygosity across camel populations. Violin plots show the distribution of FROH values, with boxplots indicating the median and interquartile range (IQR).

Kazakh camel populations (KAZ_B and KAZ_D) exhibited relatively high nucleotide diversity (θπ), indicating the maintenance of high levels of genetic variation. KAZ_B showed the highest nucleotide diversity among all populations (θπ = 1.551 × 10^−3^), followed by the Iranian (1.407 × 10^−3^) and KAZ_D (1.307 × 10^−3^) populations. In contrast, Arabian populations (Saudi Arabia, Oman, Kuwait, and Qatar) showed lower nucleotide diversity (θπ = 0.502–0.520 × 10^−3^), whereas the XJ (0.950 × 10^−3^) and NMG (0.901 × 10^−3^) populations displayed intermediate values (Figure 2B). LD decay patterns also differed among populations, with KAZ_B and KAZ_D showing intermediate LD decay rates broadly comparable to those observed in XJ and NMG, whereas the Arabian dromedary populations exhibited more rapid LD decay (Figure 2A).

Runs of homozygosity (ROHs) further revealed differences in genomic homozygosity among populations. Across all camel populations, the majority of ROHs were within the 0.5–1 Mb length category, whereas long ROHs (>4 Mb) were rare. In KAZ_D, the mean total number of ROHs was 121.133, with 100.600 in the 0.5–1 Mb category, 18.533 in the 1–2 Mb category, 2.000 in the 2–4 Mb category, and no ROHs > 4 Mb. In KAZ_B, the mean total number of ROHs was 89.500, including 78.045 in the 0.5–1 Mb category, 10.545 in the 1–2 Mb category, 0.864 in the 2–4 Mb category, and only 0.045 > 4 Mb. Significant differences were observed between KAZ_B and all Arabian populations, NMG, and XJ for 0.5–1 Mb ROHs and 1–2 Mb ROHs (all FDR-adjusted *p* < 0.05), and between KAZ_B and NMG and Oman for 2–4 Mb ROHs (*p* = 0.047 and 0.020, respectively; Table S8). In Arabian dromedary populations, the mean numbers of 2–4 Mb and >4 Mb ROHs ranged from 2.000 to 3.811 and from 0 to 0.200, respectively. KAZ_B also had a lower mean number of >4 Mb ROHs than XJ (0.059) and NMG (0.235), although these differences were not statistically significant. The predominance of short ROHs and the scarcity of long ROHs in KAZ_D and KAZ_B suggest that the observed homozygosity patterns may be more strongly influenced by older shared ancestry than by relatively recent inbreeding (Figure 2C).

Consistent with these observations, the mean ROH-based genomic inbreeding coefficient (FROH; mean ± SD) was 0.034 ± 0.027 in KAZ_B and 0.050 ± 0.017 in KAZ_D, with corresponding median values of 0.037 (95% CI: 0.016–0.043) and 0.056 (95% CI: 0.046–0.062), respectively. In Arabian dromedary populations, mean FROH values ranged from 0.059 ± 0.008 to 0.090 ± 0.061, with median values ranging from 0.058 to 0.070. The mean FROH values for XJ and NMG were 0.064 ± 0.026 and 0.079 ± 0.044, with corresponding median values of 0.054 and 0.069 (Figure 2D; Table S8). KAZ_B showed significantly lower FROH than KAZ_D, all Arabian populations, NMG, and XJ (all FDR-adjusted *p* < 0.05; Table S8). Overall, Kazakh camel populations particularly KAZ_B, exhibited relatively high genomic diversity and lower levels of genomic inbreeding compared with Arabian populations.

### 3.4. Genome-Wide Selective Sweep Analysis in Kazakh Dromedary Camels

**Figure 3:**
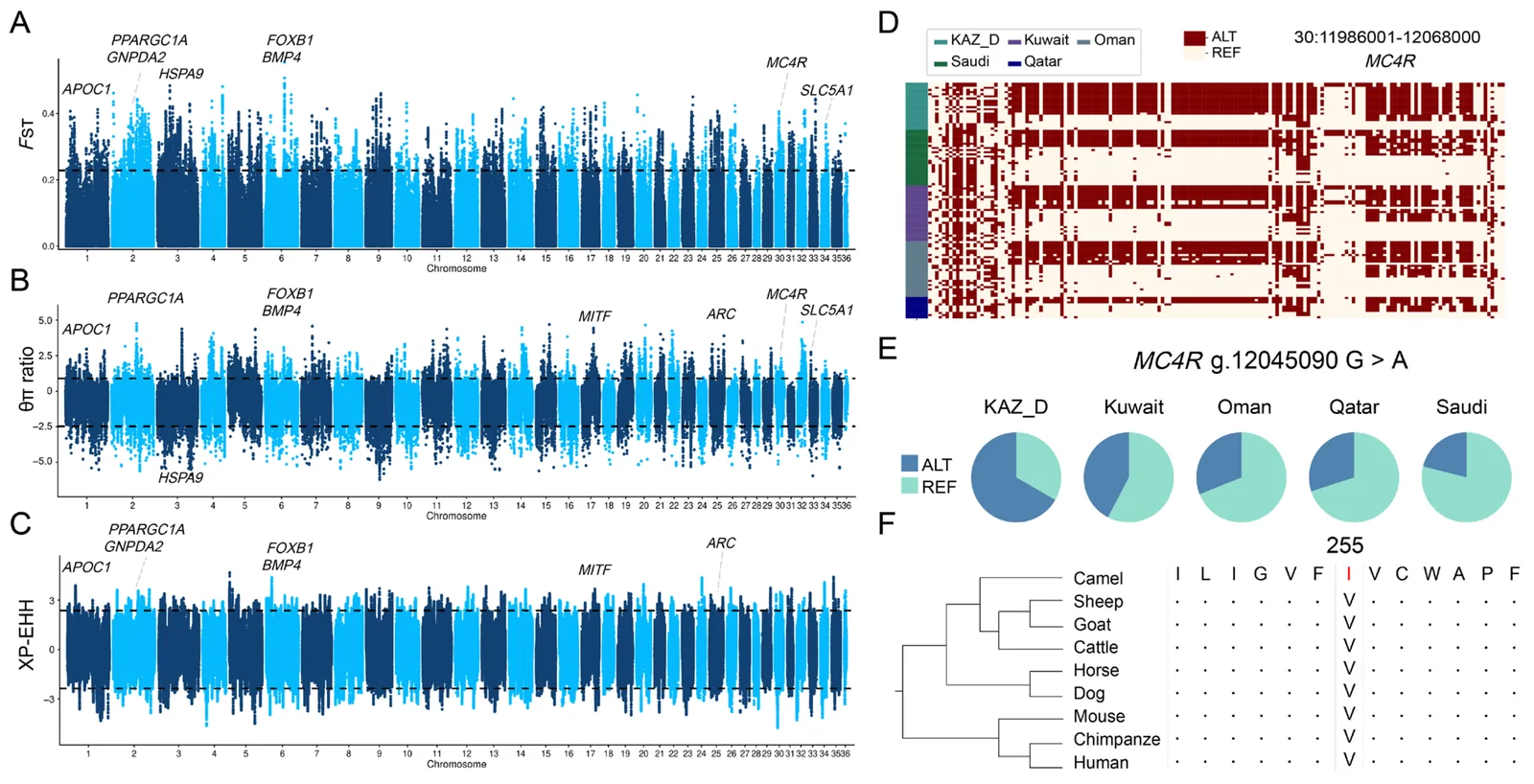
Positive selection signatures in Kazakh dromedary populations. Manhattan plots of genome-wide selection signatures based on (**A**) *F*_ST_, (**B**) θπ ratio, and (**C**) XP-EHH. (**D**) Genotype distribution across KAZ_D and Arabian dromedary populations within the *MC4R* candidate region on chromosome 30 (Chr 30: 11,986,001–12,068,000). (**E**) Distribution of alternative (ALT) and reference (REF) allele frequencies of *MC4R* variant across KAZ_D and Arabian dromedary populations. (**F**) Multiple sequence alignment of MC4R across mammalian species, showing a camel-specific amino acid substitution at the highly conserved position 255.

Genome-wide selection analysis using *F*_ST_, θπ ratio, and XP-EHH identified candidate regions within the top 1% of selection signals between KAZ_D and Saudi populations. Comparison of the candidate-gene sets identified by the three methods revealed 642 genes shared between *F*_ST_ and θπ ratio, 369 between *F*_ST_ and XP-EHH, and 703 between θπ ratio and XP-EHH, with 237 candidate genes supported by all three methods. A prominent candidate identified by the selection analyses was *MC4R*, located on chromosome 30. Furthermore, in the comparison between KAZ_D and the combined dromedary populations (Saudi, Iran, Kuwait, Oman, and Qatar), the *MC4R* gene was identified within the top 1% candidate regions by both the θπ ratio and XP-EHH analyses, further indicating that this selection signal was not restricted to the KAZ_D–Saudi comparison (Figure 3A–C and Figure S8A, and Tables S9–S11). Further examination of the candidate region (Chr30:11,986,001–12,068,000) revealed distinct patterns of alternative and reference alleles across Kazakh and Arabian dromedary populations (Figure 3D).

Further analysis identified a missense variant in *MC4R* (chr30:12045090 G>A; p.V255I). The alternative allele frequency was substantially higher in the KAZ_D (0.667) than in Arabian dromedary populations (0.211–0.423; Figure 3E), with additional variant-level information provided in Table S12. Multiple sequence alignment of the MC4R protein across mammalian species (camel, sheep, goat, cattle, horse, dog, mouse, chimpanzee, and human) revealed a high degree of conservation at amino acid position 255, with valine (V) conserved across all examined non-camel species (Figure 3F).

In addition to *MC4R*, the selection signature analyses identified several candidate genes in Kazakh dromedaries including *PPARGC1A*, *GNPDA2*, *APOC1*, *MITF*, *SLC5A1*, *FOXB1*, and *BMP4* (Figure 3A–C and Tables S9–S11). GO enrichment analysis further revealed enrichment of biological processes related to temperature homeostasis and adrenergic receptor signaling, suggesting potential involvement of neuroendocrine regulation and thermoregulatory processes in the environmental adaptation of Kazakh dromedaries (FDR < 0.05; Figure S6).

### 3.5. Genome-Wide Selective Sweep Analysis in Kazakh Bactrian Camels

**Figure 4:**
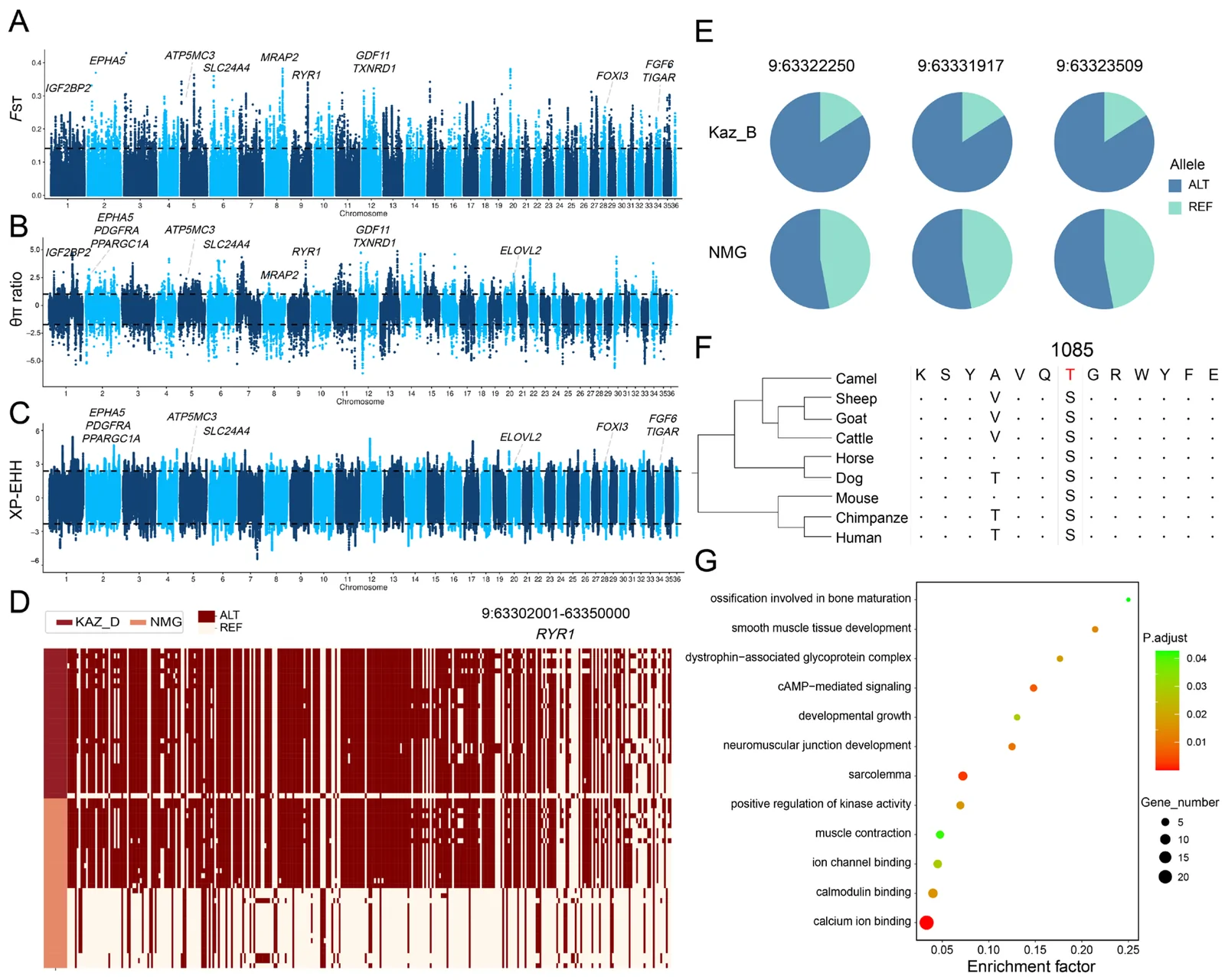
Positive selection signatures in Kazakh Bactrian populations. Genome-wide selection scans based on (**A**) *F*_ST_, (**B**) θπ ratio, and (**C**) XP-EHH. (**D**) Genotype distribution across Kazakh Bactrian (KAZ_B) and Inner Mongolian Bactrian (NMG) camel populations within the *RYR1* candidate region on chromosome 9 (Chr 9: 63,302,001–63,350,000). (**E**) Distribution of alternative (ALT) and reference (REF) allele frequencies of the three *RYR1* missense variants, chr9:63322250 (p.S1085T), chr9:63323509 (p.I1184V), and chr9:63331917 (p.I1641V), across KAZ_B and NMG camel populations. (**F**) Multiple sequence alignment of *RYR1* across mammalian species, showing a Bactrian camel-specific amino acid substitution at the highly conserved position 1085. (**G**) KEGG and GO pathway enrichment analyses of candidate genes in Kazakh Bactrian camels. Significantly enriched pathways and GO terms were defined at a corrected *p* < 0.05.

Several candidate regions were identified between KAZ_B and NMG camel populations. Comparison of the candidate-gene sets identified by the three selection methods revealed 566 genes shared between *F*_ST_ and θπ ratio, 442 between *F*_ST_ and XP-EHH, and 616 between θπ ratio and XP-EHH, with 231 candidate genes supported by all three methods. A prominent selection signal encompassing *RYR1* was identified on chromosome 9. Furthermore, in the comparison between KAZ_B and the combined domestic Bactrian camel populations (XJ and NMG), the *RYR1* gene was identified within the top 1% candidate regions by both *F*_ST_ and the θπ ratio analyses, suggesting that this selection signal was not restricted to the KAZ_B–NMG comparison (Figure 4A–C and Figure S8B, and Tables S13–S15). Further analysis revealed marked haplotype differentiation between KAZ_B and NMG populations (Figure 4D). Three missense variants in complete linkage disequilibrium (r^2^ = 1; Figure S7 and Table S17) were identified within *RYR1*: chr9:63322250 (p.S1085T), chr9:63323509 (p.I1184V), and chr9:63331917 (p.I1641V) (Figure 4E). The alternative allele frequencies of the three *RYR1* variants were higher in KAZ_B (0.841) than in NMG (0.529); additional variant-level information is provided in Table S16.

Multiple sequence alignment of *RYR1* across mammalian species revealed that serine (S) at amino acid position 1085 is highly conserved, indicating the potential functional significance of the p.S1085T substitution (Figure 4F). Furthermore, multiple candidate genes were identified within genomic regions under positive selection, including *PPARGC1A*, *ATP5MC3*, *IGF2BP2*, *ELOVL2*, *FGF6*, *UCP1*, *PDGFRA*, *MRAP2*, *SLC24A4*, *TXNRD1*, *TIGAR*, *GDF11* and *EPHA5* (Figure 4A–C, Tables S13–S15). These candidate genes were distributed across several biological categories related to mitochondrial energy metabolism, skeletal muscle function, fatty acid metabolism, oxidative stress response, calcium homeostasis, and nervous system development.

GO enrichment analysis revealed that candidate genes were significantly enriched in terms related to calcium ion binding, muscle contraction, neuromuscular junction development, smooth muscle tissue development, cAMP-mediated signaling, and ossification involved in bone maturation (FDR < 0.05; Figure 4G).

## 4. Discussion

In this study, we combined newly generated whole-genome sequencing data from Kazakh dromedary and Bactrian camels with publicly available genomes to comprehensively characterize the population structure, genetic diversity, and genomic signatures of adaptation across domestic camel populations. Our findings provide new insights into the genetic relationships, diversity patterns, and genomic differentiation of Kazakh camels and suggest that their present-day genomic diversity may reflect the combined influences of demographic history, breeding practices and associated artificial selection, and environmental selection.

The population structure analyses demonstrated clear genetic differentiation between dromedary and Bactrian camels, consistent with their long-term evolutionary divergence and independent domestication histories. Nevertheless, Kazakh camel populations exhibited more complex patterns of shared genetic ancestry than Arabian dromedary populations. These patterns may reflect multiple demographic and breeding processes. In Central Asia, dromedaries and Bactrian camels have coexisted for centuries, and intentional hybridization has been widely practiced to combine desirable production traits, including higher milk yield, greater body size, improved draft capacity, and adaptation to harsh continental environments. Historical crossbreeding has produced well-known hybrids such as Nar and Nar-Maya, which remain widely used in Kazakhstan [16,22].

Our findings are consistent with previous genomic studies demonstrating that hybridization has played a major role in shaping the genetic diversity of Central Asian camel populations. For example, Ming et al. [8] reported that domestic Bactrian camels from central Asia possess higher genetic diversity than East Asian populations, largely attributable to historical introgression from dromedaries. Similarly, recent whole-genome analyses of Kazakh hybrid camels confirmed extensive admixture and showed that hybrids occupy intermediate genetic positions between the parental species [22]. In contrast, Arabian dromedary populations displayed relatively homogeneous ancestry, which agrees with previous whole-genome studies reporting limited population subdivision across the Arabian Peninsula despite minor geographic differentiation [42]. The relatively homogeneous genetic background of Arabian camels likely reflects more geographically restricted breeding systems and lower levels of interspecific hybridization than those observed in Central Asia. This complex demographic and breeding history may also help explain the intermediate LD decay observed in KAZ_B and KAZ_D, despite their relatively high nucleotide diversity, as LD patterns can be influenced by population structure, admixture, and historical gene flow in addition to genetic diversity. Together, these results indicate that domestication history, hybrid breeding, and regional gene flow have been major drivers shaping the present-day genomic structure of domestic camel populations.

The strong selection signal encompassing *MC4R* in Kazakh dromedaries is particularly noteworthy given its central role in energy homeostasis and metabolic regulation. *MC4R* encodes the melanocortin-4 receptor, a member of the G protein-coupled receptor (GPCR) family that plays a central role in regulating feeding behavior, energy metabolism, and body weight homeostasis. Recent studies have demonstrated that MC4R not only regulates appetite but also contributes to whole-body energy balance through modulation of receptor signaling and receptor oligomerization [43]. In addition, persistent ubiquitination of *MC4R* promotes its dynamic trafficking within the primary cilium, thereby maintaining the central regulation of body weight [44]. Previous studies have further established the critical role of *MC4R* in maintaining energy homeostasis, regulating lipid metabolism, and controlling body weight in mammals [45]. Notably, *MC4R* signaling has also been implicated in thermogenic responses to cold through the regulation of energy expenditure, glucose metabolism, and the browning of white adipose tissue via the autonomic nervous system [46]. In the present study, the substantially higher frequency of the p.V255I alternative allele in Kazakh dromedaries (0.667) compared with Arabian dromedary populations (0.211–0.423), together with evolutionary conservation of amino acid position 255 across mammals, provides additional evidence that this locus may have been subject to differential selection between populations inhabiting contrasting climatic environments. Taken together, these findings identify *MC4R* as a candidate gene potentially associated with metabolic and thermoregulatory responses in Kazakh dromedary camels. However, additional functional validation is required to confirm its role.

Besides *MC4R*, several candidate genes support the hypothesis that energy metabolism has been an important target in Kazakh dromedaries. *PPARGC1A* (PGC-1α) encodes a key transcriptional coactivator involved in mitochondrial biogenesis and fatty acid oxidation and plays a central role in maintaining oxidative metabolism and energy homeostasis [47]. *GNPDA2* is involved in the hexosamine metabolic pathway and has been implicated in the regulation of glucose and lipid metabolism [48], whereas *APOC1*, a member of the apolipoprotein family, contributes to lipoprotein metabolism and cholesterol transport and is therefore important for maintaining lipid homeostasis [49]. Furthermore, *MITF* may enhance mitochondrial metabolic capacity by activating the PGC1α–PPARγ-fatty acid oxidation (FAO) pathway, thereby promoting lipid utilization and adaptive metabolic remodeling [50].

Other candidate genes point to potential involvement in neural regulation and development processes. *ARC*, a neuronal immediate-early gene, plays an important role in synaptic plasticity and long-term memory formation [51]. *FOXB1* is involved in organ morphogenesis and diencephalic neural development in mice [52], while *BMP4* plays an essential role in normal embryonic and cardiac development in humans [53]. Although the direct contribution of these genes to climatic adaptation in camels remains to be established, their occurrence within candidate regions under selection suggests that adaptive differentiation in Kazakh dromedaries may involve not only metabolic pathway but also broader neural and developmental regulatory processes.

In contrast to the selection signatures observed in Kazakh dromedaries, a selection signal associated with *HSPA9* was detected in the Saudi population. *HSPA9* encodes a mitochondrial molecular chaperone, and previous studies have demonstrated its important anti-apoptotic role under heat-stress conditions [54]. Given the prolonged exposure of Saudi Arabian dromedaries to high ambient temperature, selection involving *HSPA9* may contribute to cellular protection against thermal stress and potentially facilitate adaptation to hot environments. The contrasting selection patterns observed between Kazakh and Saudi dromedaries may therefore reflect population-specific responses to different environmental conditions, although demographic history and breeding practices may also have contributed to these genomic differences.

In the present study, *RYR1* exhibited marked haplotype differentiation between Kazakh Bactrian and Inner Mongolian Bactrian camel populations and harbored three completely linked missense variants (p.S1085T, p.I1184V, and p.I1641V). Notably, serine at position 1085 was highly conserved across the examined mammalian species, suggesting that the p.S1085T substitution may have potential functional significance. Functional enrichment analysis further revealed significant enrichment of calcium signaling-related pathways involving *RYR1*, highlighting the potential importance of Ca^2+^-dependent processes in the selected genomic regions. The selection signal encompassing *RYR1* in Kazakh Bactrian camels is of particular interest given its reported role in skeletal muscle function and thermogenesis. *RYR1* encodes ryanodine receptor 1, the major Ca^2+^-release channel of the skeletal muscle sarcoplasmic reticulum and key regulator of excitation–contraction coupling, which is essential for skeletal muscle contraction [55,56].

Beyond its fundamental role in muscle contraction, RYR1-mediated Ca^2+^ leak has been reported to promote skeletal muscle non-shivering thermogenesis, thereby increasing heat production and contributing to the maintenance of body temperature [57]. Given the extremely cold continental climate experienced by Kazakh Bactrian camels, selection at the *RYR1* locus may be associated with skeletal muscle thermogenesis and energy expenditure under low-temperature conditions. Moreover, because RYR1-mediated Ca^2+^ release is central to excitation–contraction coupling and skeletal muscle function [58], variation at this locus may potentially contribute to sustained muscular activity during long-distance locomotion. However, these interpretations remain hypothetical, and further functional and phenotypic validation is required to determine the biological effects of *RYR1* variants in camels.

In addition to *RYR1*, several candidate genes with potential roles in energy and skeletal muscle metabolism were identified in Kazakh Bactrian camels. *PPARGC1A* (PGC-1α) encodes a key transcriptional coactivator involved in mitochondrial biogenesis and fatty acid oxidation, thereby promoting oxidative metabolism and contributing to exercise endurance [47,59]. *ATP5MC3* encodes a membrane subunit of mitochondrial ATP synthase (complex V), an essential component of oxidative phosphorylation and ATP production [60]. IGF2BP2 is an RNA-binding protein dynamically expressed in skeletal muscle and has been implicated in the post-transcriptional regulation of multiple metabolic pathways, including glycolysis, glutamine metabolism, and lipid metabolism [61,62,63,64].

Several additional candidate genes are associated with cold adaptation and lipid metabolism. *ELOVL2* encodes a rate-limiting enzyme responsible for the biosynthesis of long-chain polyunsaturated fatty acids [65]. *FGF6* promotes thermogenesis by inducing *UCP1* expression [66], whereas *PDGFRA* regulates adipocyte progenitor maintenance, adipogenesis, and adipose tissue remodeling [67]. *MRAP2* modulates appetite and energy balance through regulation of the MC4R signaling pathway [68].

Other candidate genes may contribute to cellular homeostasis under environmental stress. *SLC24A4* (NCKX4) functions as a potassium-dependent Na^+^/Ca^2+^ exchanger involved in calcium homeostasis and oxidative stress responses [69,70]. *TXNRD1* participates in cellular redox metabolism and antioxidant defense [71], whereas *TIGAR* protects cells by suppressing glycolysis and reducing reactive oxygen species production [72]. Finally, *GDF11* and *EPHA5* are associated with nervous system development and function. *GDF11* contributes to neural maintenance and delays age-related neuronal decline [73], whereas *EPHA5* plays important roles in neural development and cell–cell communication [74]. These genes may contribute to sensory and motor coordination required for movement and navigation across the complex steppe and desert environments inhabited by Kazakh Bactrian camels.

A limitation of this study is the use of the dromedary CamDro3 reference genome for all camel species. Although a common reference and uniform bioinformatics pipeline ensured a consistent genomic coordinate system across populations, reference-genome divergence may introduce mapping and variant-calling biases in domestic and wild Bactrian camels and may consequently influence estimates of nucleotide diversity, ROHs, and selection signals. In addition, sample sizes were uneven among populations, with some populations represented by relatively few individuals. This sampling imbalance may increase uncertainty in population-level estimates and potentially affect comparisons of genetic diversity among populations. Therefore, cross-species comparisons should be interpreted with caution.

Overall, our findings reveal distinct patterns of genomic differentiation and candidate selection signals in Kazakh dromedary and Bactrian camels. Kazakh dromedaries showed candidate signals involving genes related to energy metabolism and thermoregulation, whereas Kazakh Bactrian camels showed signals involving genes associated with calcium signaling, skeletal muscle function, and mitochondrial energy metabolism. These patterns may be relevant to physiological responses to the continental environment of Kazakhstan. However, demographic history, genetic drift, gene flow, and artificial selection associated with breeding practices may also have contributed to the observed genomic differentiation. Therefore, the identified candidate genes should be considered hypotheses for potential environmental adaptation, and further functional and phenotypic validation is required to confirm their adaptive significance.

## 5. Conclusions

This study provides a comprehensive genomic analysis of Kazakh dromedary and Bactrian camels using newly generated and publicly available whole-genome sequencing data. Kazakh camel populations exhibited higher genomic diversity, lower genomic inbreeding, and distinct population structure compared with Arabian camel populations, patterns that may reflect differences in demographic history, gene flow, and regional breeding practices. Genome-wide selection analysis identified *MC4R* as a prominent candidate gene in Kazakh dromedaries, potentially related to energy balance, metabolic regulation, and thermoregulatory processes, whereas *RYR1* was identified in Kazakh Bactrian camels, with potential roles in calcium signaling, skeletal muscle function, and thermogenesis. These findings provide insights into genomic variation potentially associated with environmental adaptation in Kazakh camels and valuable genomic resources for future studies of camel evolution, conservation, and molecular breeding. However, further functional and phenotypic validation is required to confirm the roles of these candidate genes in climatic adaptation.

## Data Availability

The raw sequencing data are available in the NCBI Sequence Read Archive (SRA) under BioProject accession number PRJNA1480395.

## Supplementary Materials

The following supporting information can be downloaded at https://www.mdpi.com/article/10.3390/ani16172793/s1, Figure S1: Representative images of Bactrian and dromedary camels from Kazakhstan; Figure S2: Principal component analysis (PCA) of domestic camel populations from different geographical regions, excluding wild Bactrian camels; Figure S3: Principal component analysis (PCA) of domestic camel populations by species; Figure S4: ADMIXTURE model-based clustering analysis of domestic camel populations assuming different numbers of ancestral components (*K* = 2–9) based on SNPs; Figure S5: Cross-validation error (CV) estimates for ADMIXTURE analyses based on SNPs retained after LD pruning; Figure S6: Gene Ontology (GO) enrichment analyses of candidate genes identified in the comparison between Kazakh and Saudi Arabian dromedary camel populations; Figure S7: Linkage disequilibrium (LD) blocks in the *RYR1* candidate region identified from the selection analysis between Kazakh and Inner Mongolian Bactrian camel populations; Figure S8: *MC4R* and *RYR1* identified within the top 1% candidate regions in additional combined-population comparisons; Table S1: Summary of sequencing data from local camels in Kazakhstan; Table S2: Sample information and sequencing quality metrics for publicly available camel genomes used in this study; Table S3: Summary of sequencing, mapping, and genomic quality metrics across camel populations and species; Table S4: Pairwise kinship analysis of camel individuals; Table S5: Individual ancestry proportions estimated by ADMIXTURE at *K* = 3; Table S6: Median nucleotide diversity (θπ) across camel populations with 95% bootstrap confidence intervals; Table S7: Individual-level summary of autosomal runs of homozygosity (ROH) and ROH-based genomic inbreeding coefficients (FROH) in camels; Table S8: Comprehensive summary of autosomal ROH length distribution, FROH descriptive statistics, and pairwise comparisons between Kazakh and other camel populations; Table S9: Top 1% candidate genes identified by *F*_ST_ in *Camelus dromedarius* (KAZ_D vs Saudi); Table S10: Top 1% candidate genes identified by the θπ ratio in *Camelus dromedarius* (KAZ_D vs Saudi); Table S11: Top 1% candidate genes identified by XP-EHH in Camelus dromedarius (KAZ_D vs. Saudi); Table S12: Quality metrics, genotype counts, and allele frequencies of the *MC4R* missense variant (chr30:12045090 G>A, p.V255I); Table S13: Top 1% candidate genes identified by *F*_ST_ in *Camelus bactrianus* (KAZ_B vs. NMG); Table S14: Top 1% candidate genes identified by θπ ratio in *Camelus bactrianus* (KAZ_B vs. NMG); Table S15: Top 1% candidate genes identified by XP-EHH in *Camelus bactrianus* (KAZ_B vs. NMG); Table S16: Quality metrics, genotype counts, and allele frequencies of three completely linked *RYR1* missense variants (chr9:63322250 G>C, p.S1085T; chr9:63323509 A>G, p.I1184V; and chr9:63331917 A>G, p.I1641V); Table S17: Pairwise linkage disequilibrium (LD) between SNPs (r^2^ values).

## Abbreviations

The following abbreviations are used in this manuscript:

KAZ_B	Kazakh Bactrian camels
KAZ_D	Kazakh dromedary camels
XJ	Xinjiang Bactrian camels
NMG	Inner Mongolian Bactrian camels
ALT	Alternative allele
BAM	Binary Alignment/Map
bp	Base pair
CV	Cross-validation
DNA	Deoxyribonucleic acid
FDR	False discovery rate
FROH	Genomic inbreeding coefficient based on runs of homozygosity
*F*_ST_	Fixation index
GATK	Genome Analysis Toolkit
GO	Gene Ontology
gVCF	Genomic Variant Call Format
KEGG	Kyoto Encyclopedia of Genes and Genomes
LD	Linkage disequilibrium
NJ	Neighbor-joining
PCA	Principal component analysis
REF	Reference allele
ROH	Runs of homozygosity
SAM	Sequence Alignment/Map
SNP	Single-nucleotide polymorphism
VCF	Variant Call Format
WGS	Whole-genome sequencing
XP-EHH	Cross-population extended haplotype homozygosity
θπ	Nucleotide diversity
