# Supplementary material for: Whole-Genome Sequencing Reveals Population Structure, Genetic Diversity, and Selection Signatures in Kazakh Dromedary and Bactrian Camels
Source: Animals (Basel). 2026 Sep 5;16(17):2793. doi: 10.3390/ani16172793 (PMC13564503; doi:10.3390/ani16172793)
Supplement: Supplementary file 1 [file animals-16-02793-s001.zip › animals-4508492-supplementary/Supplementary Figures S1-S8.pdf]

Supplementary figures

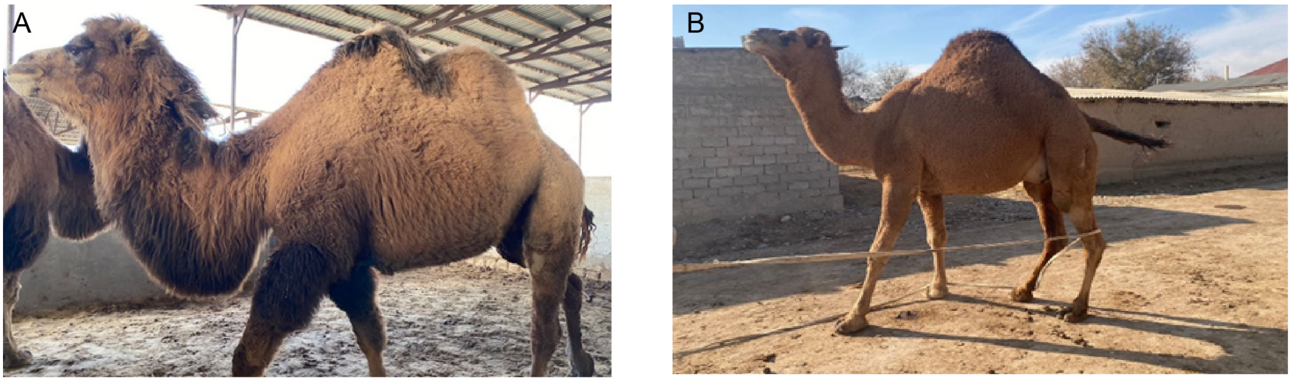

Figure S1. Representative images of Bactrian and dromedary camels from Kazakhstan. (A) Bactrian camel and (B) dromedary camel, illustrating the characteristic external morphology of each camel species. Photographs by Yessengali Ussenbekov (Faculty of Veterinary Medicine, Kazakh National Agrarian Research University).

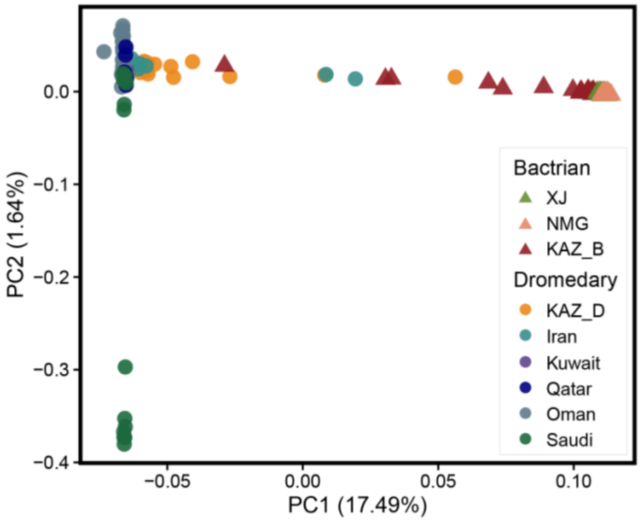

Figure S2. Principal component analysis (PCA) of domestic camel populations from different geographical regions, excluding wild Bactrian camels.

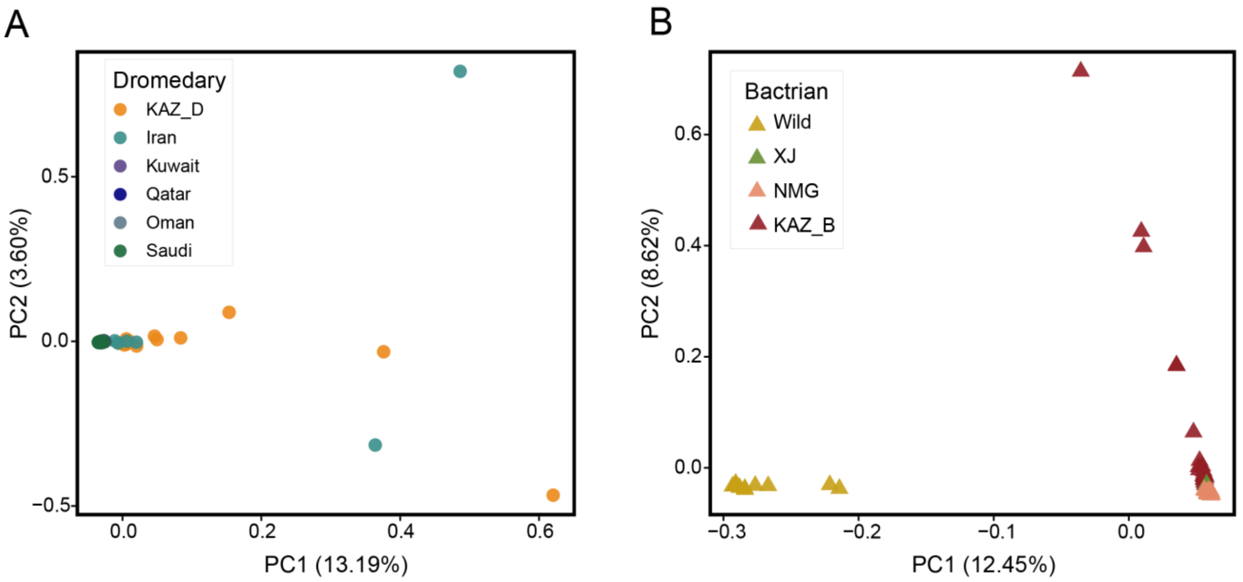

Figure S3. Principal component analysis (PCA) of domestic camel populations by species. (A) PCA of domestic dromedary populations; (B) PCA of domestic Bactrian camel populations.

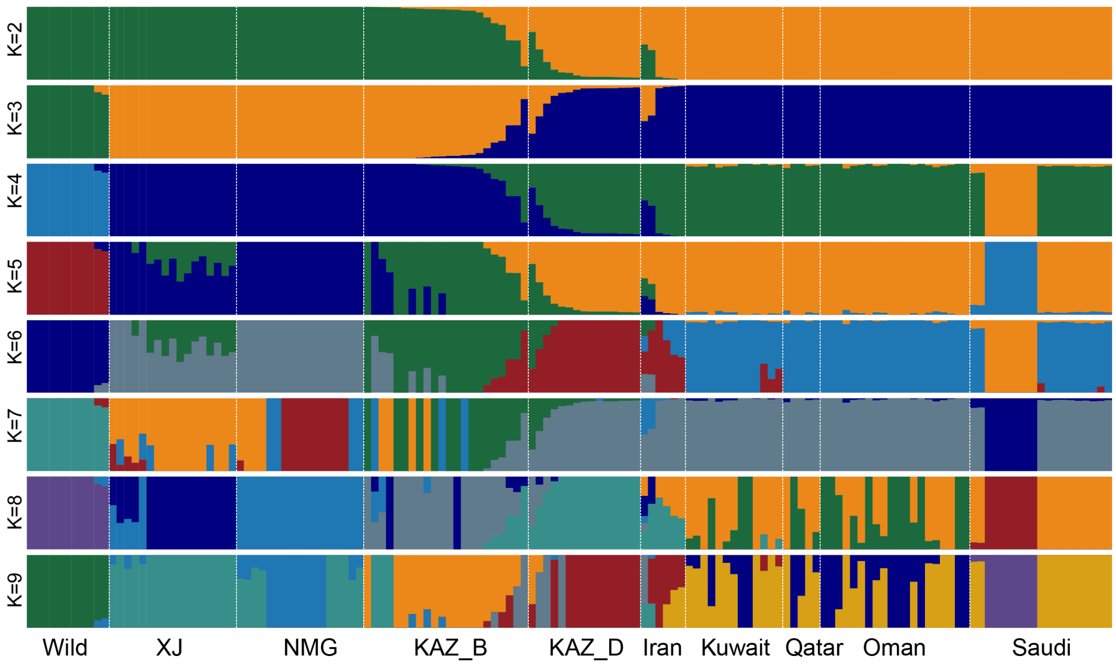

Figure S4. ADMIXTURE model-based clustering analysis of domestic camel populations assuming different numbers of ancestral components ($K = 2–9$) based on SNP data.

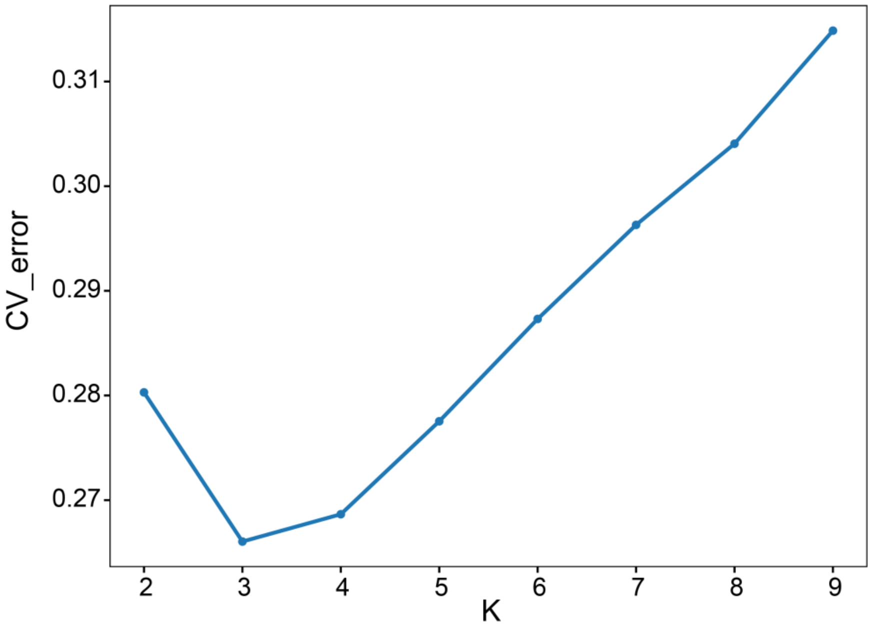

Figure S5. Cross-validation error (CV) estimates for ADMIXTURE analyses based on SNPs retained after LD pruning. The lowest CV error was observed at $K = 3$.

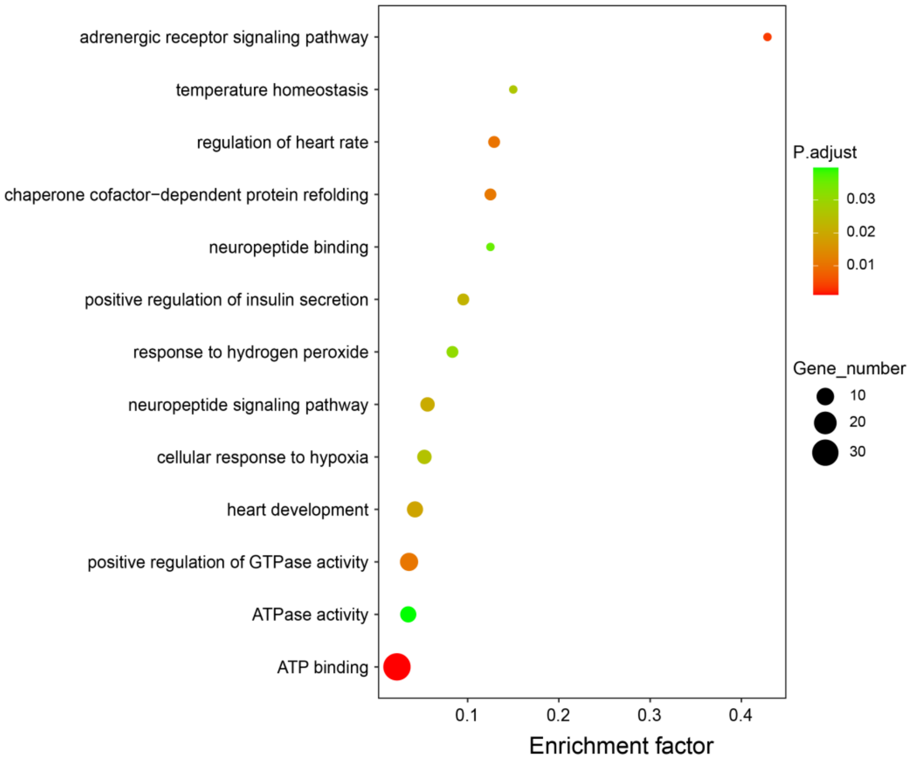

Figure S6. Gene Ontology (GO) enrichment analysis of candidate genes identified in the comparison between Kazakh and Saudi Arabian dromedary camel populations.

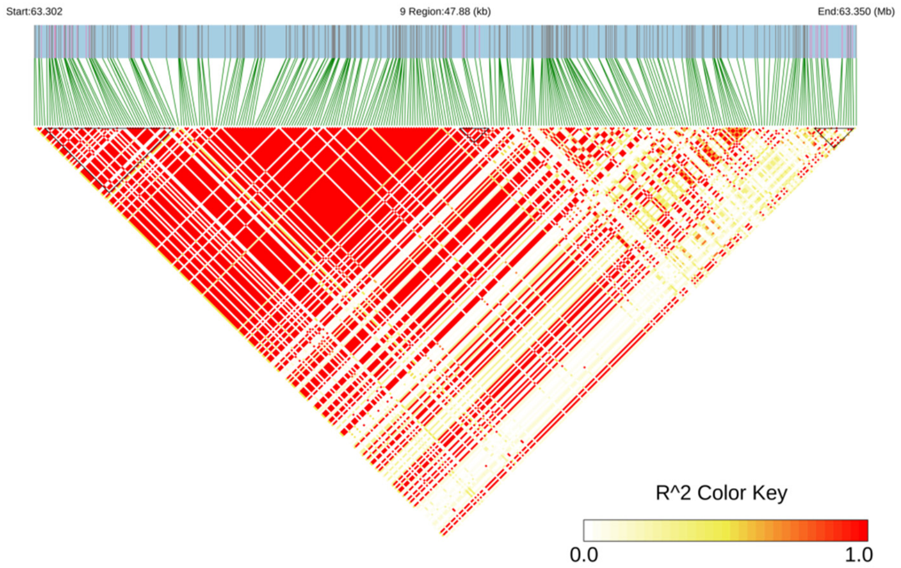

Figure S7. Linkage disequilibrium (LD) blocks in the *RYR1* candidate region identified from the selection analysis between Kazakh and Inner Mongolian Bactrian camel populations.

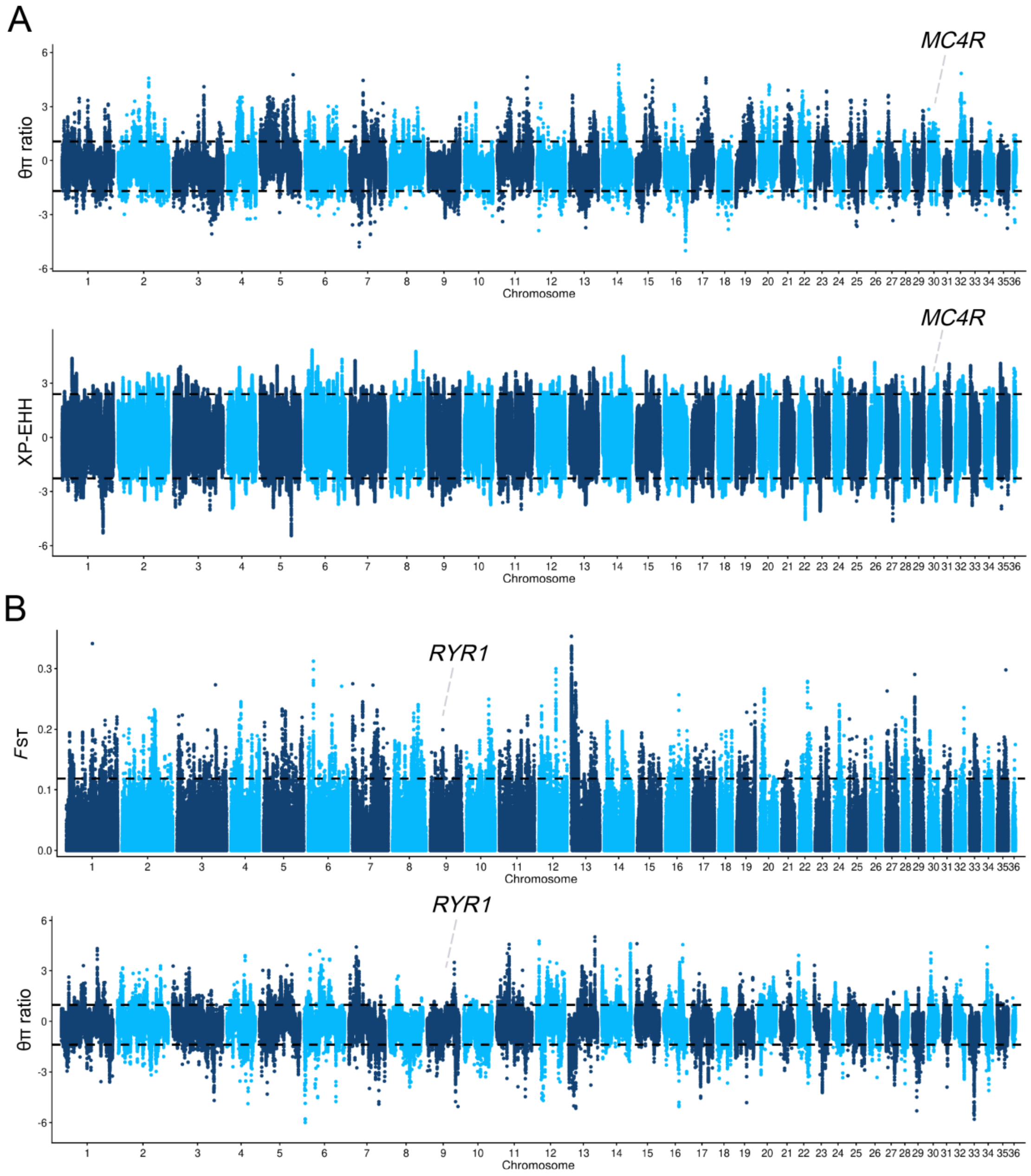

Figure S8. *MC4R* and *RYR1* identified within the top 1% candidate regions in additional combined-population comparisons. (A) *MC4R* was identified within the top 1% candidate regions detected using the θπ ratio and XP-EHH in the comparison between KAZ_D and the combined dromedary populations (Saudi, Iran, Kuwait, Oman, and Qatar). (B) *RYR1* was identified within the top 1% candidate regions detected using F_{ST} and the θπ ratio in the comparison between KAZ_B and the combined domestic Bactrian camel populations (NMG and XJ).
